# Identification of an inflammatory response signature associated with prognostic stratification and drug sensitivity in lung adenocarcinoma

**DOI:** 10.1038/s41598-022-14323-6

**Published:** 2022-06-16

**Authors:** Congkuan Song, Zilong Lu, Kai Lai, Donghang Li, Bo Hao, Chenzhen Xu, Shize Pan, Ning Li, Qing Geng

**Affiliations:** Department of Thoracic Surgery, Renmin Hospital of Wuhan University, No.238 Jiefang Road, Wuchang District, Wuhan, 430060 China

**Keywords:** Cancer, Computational biology and bioinformatics, Drug discovery, Immunology, Biomarkers, Diseases, Oncology, Risk factors

## Abstract

Increasing evidence has confirmed the close connection between inflammatory response and tumorigenesis. However, the relationship between inflammatory response genes (IRGs) and the prognosis of lung adenocarcinoma (LUAD) as well as the response to drug therapy remains poorly investigated. Here, we comprehensively analyzed IRGs RNA expression profiling and clinical features of over 2000 LUAD patients from 12 public datasets. The Cox regression method and LASSO analysis were combined to develop a novel IRG signature for risk stratification and drug efficacy prediction in LUAD patients. Enriched pathways, tumor microenvironment (TME), genomic and somatic mutation landscape in different subgroups were evaluated and compared with each other. This established IRG signature including 11 IRGs (ADM, GPC3, IL7R, NMI, NMURI, PSEN1, PTPRE, PVR, SEMA4D, SERPINE1, SPHK1), could well categorize patients into significantly different prognostic subgroups, and have better predictive in independently assessing survival as compared to a single clinical factor. High IRG scores (IRGS) patients might benefit more from immunotherapy and chemotherapy. Comprehensive analysis uncovered significant differences in enriched pathways, TME, genomic and somatic mutation landscape between the two subgroups. Additionally, integrating the IRGS and TNM stage, a reliable prognostic nomogram was developed to optimize survival prediction, and validated in an independent external dataset for clinical application. Take together, the proposed IRG signature in this study is a promising biomarker for risk stratification and drug efficacy prediction in LUAD patients. This study may be meaningful for explaining the responses of clinical therapeutic drugs and providing new strategies for administrating sufferer of LUAD.

## Introduction

Lung adenocarcinoma (LUAD) has become the most prevalent subtype of non-small cell lung cancer (NSCLC)^1^. Despite advances in treatment methods, the prognosis of LUAD patients is far from being satisfactory. Clinically, the TNM staging system is still most widely used to judge patient prognosis, but tumor heterogeneity may make this prognostic judgment tool based on anatomical factors alone not always accurately predict patient prognosis. In addition, another headache is that due to the biological characteristics of LUAD and individual heterogeneity, patients at the same stage tend to present completely different responses to the same drug treatment, which undoubtedly brings challenges to the treatment of LUAD. Nevertheless, the treatment pattern of patients is mainly based on the tumor stage. Simple and mechanical division of patients into a certain stage will inevitably affect their prognostic judgment and treatment decisions. Thus, in the era of precision medicine, clinical practice requires a classifier to accurately distinguish between subgroups of patients with different prognosis and therapeutic responses.

**Figure 1:**
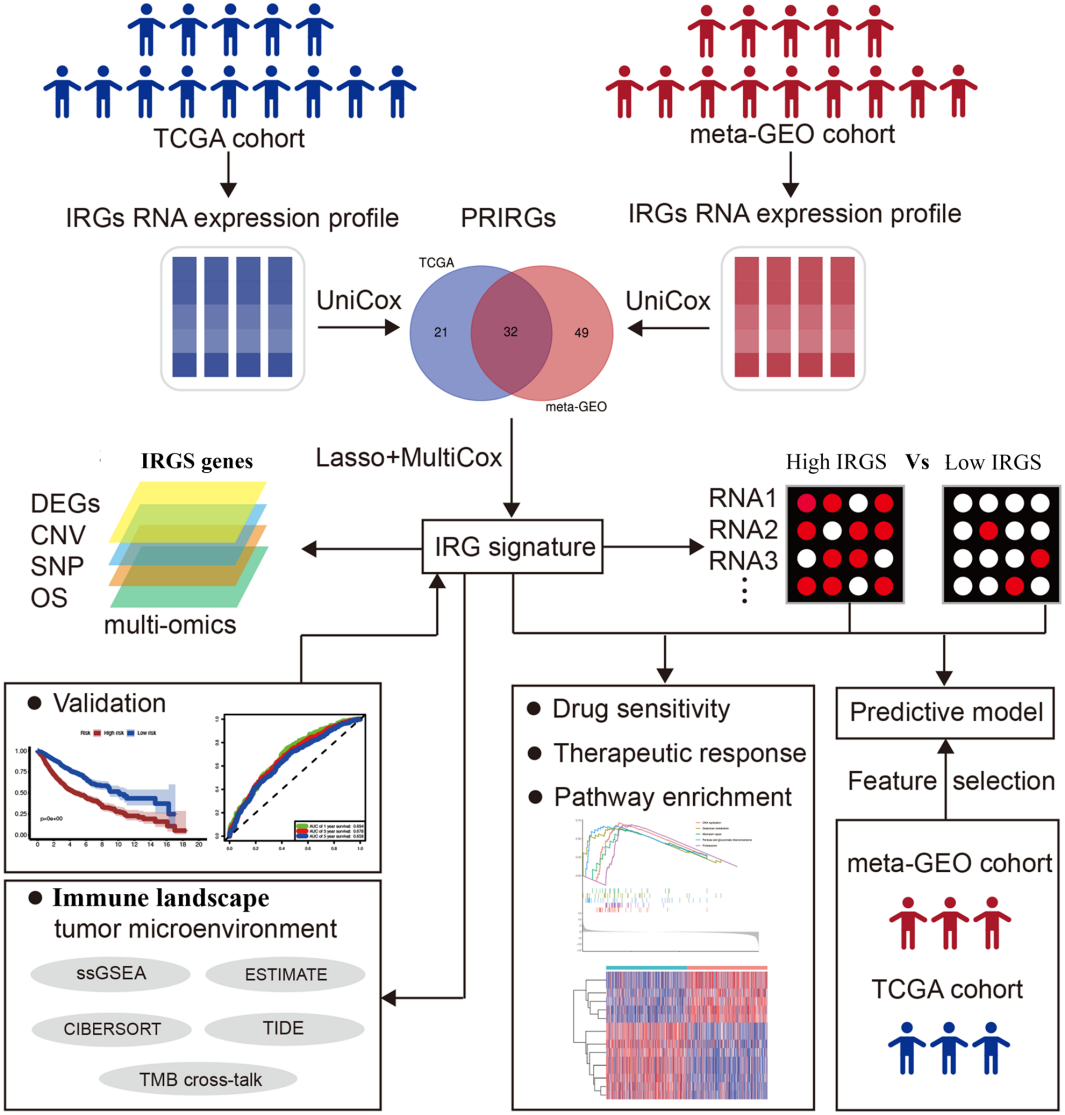
Schematic diagram of the study design.

Recent years have seen a proliferation of researches on constructing genomic signatures for risk stratification in NSCLC patients^2–10^. However, most of these prognostic signatures still faced some limitations for routine clinical practice due to limited sample sizes and low reproducibility. Previous studies have shown that the inflammatory microenvironment as the seventh hallmark of cancer could be activated to promote tumorigenesis^11–13^. These studies of Zhao et al.^14^ and Loza et al.^15^ revealed the relationship between IRGs and tumor progression as well as patient prognosis. Currently, there is no data regarding a reliable model including some IRGs to predict prognosis and drug therapy response for patients with LUAD. In this study, based on 1615 LUAD patient data from multiple microarrays, we developed a novel IRG signature including 11 genes for risk stratification and efficacy prediction, which were well validated in an independent TCGA cohort containing 500 patients. We further investigated the immune landscapes, biological pathways, as well as drug sensitivity between different subgroups. Additionally, we also established a novel prognostic nomogram to optimize survival prediction in LUAD patients, and validated it in an independent external dataset for clinical application. The workflow of this study was summarized in Fig. 1. Overall, the present study might inform accurate prognostic prediction and important treatment strategies for LUAD patients.

## Materials and methods

### Data acquisition and processing

The IRGs RNA expression profiles and clinical features of LUAD patients from 12 public cohorts were retrospectively and comprehensively analyzed. And these 12 datasets information were visible in Table S1. 200 IRGs were collected from the GSEA website (http://www.gsea-msigdb.org/gsea/index.jsp) (Table S2). A thorough query of the LUAD dataset was performed in the Gene Expression Omnibus (GEO) database in order to obtain as many and eligible samples as possible, and a total of 11 independent LUAD study datasets (GSE10072^16^, GSE14814^17^, GSE29013^18^, GSE30219^19^, GSE31210^20^, GSE37745^21^, GSE40791^22^, GSE42127^7,23^, GSE50081^2^, GSE68465^24^ and GSE72094^25^) were finally included in our study, with GSE10072, GSE30219, GSE31210, GSE40791 and GSE68465 containing corresponding lung normal tissues, and GSE14814, GSE29013, GSE30219, GSE31210, GSE37745, GSE42127, GSE50081, GSE68465 and GSE72094 containing survival data. According to the corresponding annotation files, we converted the probes to gene symbols. For genes with multiple probe set signals, their values were averaged to generate a single expression value. To reduce the batch effects from non-biological technical biases, we finally integrated them into a meta-GEO cohort using the ‘ComBat’ algorithm of ‘sva’ R package^26,27^. As an independent validation dataset, TCGA-LUAD RNA-seq data (FPKM format) were obtained from the Genomic Data Commons (GDC, https://portal.gdc.cancer.gov/). TCGA somatic mutation data were also collected from the GDC. Additionally, we also downloaded the copy number variation (CNV) profiles from UCSC Xena (https://xenabrowser.net). The CNV landscape of the key genes in human chromosomes was visualized using ‘Rcircos’ R package.

### Establishment and validation of an IRG signature

After removing samples without clear survival information, eventually, 1615 patients were included in the meta-GEO cohort, and 500 patients were included in the TCGA cohort (Table 1). Univariate Cox analysis were respectively performed for the IRGs in the two LUAD cohorts (Table S3). The common genes from the statistically significant genes (p value < 0.05) in the two cohorts, were considered prognostic-related IRGs (PRIRGs), and were then fitted into LASSO regression analysis (using ‘glmnet’ R package) to reduce the dimensionality of the data. Next, using ‘survival’ R package, multivariate Cox analysis was performed to calculate the coefficient of each gene. Subsequently, IRG scores (IRGS) was generated according to the following formula: IRGS = β1 × expression***G1*** + β2 × expression***G2*** + ⋯ + βn × expression***Gn***, where β***n*** represented the coefficient of gene***n*** and expression***Gn*** was the expression level of gene***n***. With the median IRGS as cut-off, patients were classified into high- and low-IRGS groups. Probability of survival was estimated by the Kaplan–Meier method, with differences between two groups tested using the log-rank test. The IRG signature constructed in this study was compared to these signatures constructed in other publications^28–30^. The receiver operating characteristic (ROC) curve, a combination of sensitivity (true positive rate) and specificity (true negative rate), was used to assess prediction performance of the IRGS, and the corresponding area under the curve (AUC) values were also calculated. Additionally, subgroup analysis was also performed, and the Kaplan–Meier survival curves were plotted to verify the predictive performance in the different subgroups.

**Table 1:** Basic clinicopathologic features of LUAD patients in the two cohorts.

Characteristics	Subsets	meta-GEO cohort (n = 1615)	TCGA cohort (n = 500)
Age (years)	≤ 65	809 (50.1)	237 (47.4)
	> 65	806 (49.9)	253 (50.6)
	NA	0 (0.0)	10 (2.0)
Sex	Female	811 (50.2)	270 (54.0)
	Male	804 (49.8)	230 (46.0)
TNM_stage	I and II	1423 (88.1)	392 (78.4)
	III and IV	192 (21.9)	108 (21.6)
IRGS	High	807 (50.0)	250 (50.0)
	Low	808 (50.0)	250 (50.0)

### Cell culture, and qPCR analysis

Two LUAD cell lines (A549 and H1975), and normal epithelial cell line of the human lung (beas-2B) were purchased from the Chinese Academy of Sciences in Shanghai. All cells were cultured in RPMI 1640 medium (Invitrogen, Shanghai, China) which supplemented with 10% FBS (Gibco, USA), 100 U/ml penicillin and 100 mg/ml streptomycin in a humidifed atmosphere with 5% CO2 at 37 ℃. Applying the Trizol method, the total RNA was extracted and subsequently used to synthesize cDNA and subjected to PCR reactions (all experimental procedures were performed strictly according to the instructions of the kit). GAPDH was used as the reference gene and relative gene expression was calculated by the 2−ΔΔCT method. Primer sequences of NMI, GPC3 and GAPDH were listed as following: NMI forward: 5′-AGGAGTCAGATTCCAGGTTTATGT-3′; reverse: 5′-ATCTTGTCAGCCACTCCAATCTC-3′; GPC3 forward: 5′-GCAAGTTATGTGCCCATTCTCAA-3′; reverse: 5′-TTCCAGCAAAGGGTGTCGTT-3′; GAPDH forward: 5′‐CTGTTCGACAGTCAGCCGCATC‐3′, GAPDH reverse: 5′‐GCGCCCAATACGACCAAATCCG‐3′.

### Gene set variation analysis and gene set enrichment analysis

As a non-parametric and unsupervised method, gene set variation analysis (GSVA) bypassed traditional methods of explicitly modeling phenotypes in enrichment scoring algorithms^31^, could be used to estimate changes in the activity of pathways and biological processes in expression data set samples. In this study, GSVA method was also performed to investigate the difference on biological processes between different IRGS subgroups. Additionally, gene set enrichment analysis (GSEA) was also conducted to explore whether biological functions and process were significantly different between different IRGS subgroups using ‘clusterProfiler’ R package^32^. The gene sets of ‘c5.all.v7.0.symbols’ and ‘c2.cp.kegg.v7.2.symbols’ were downloaded from MSigDB (http://www.gsea-msigdb.org/gsea/msigdb).

### Estimation of tumor microenvironment (TME)

To better characterize the LUAD immune landscape, we used a single-sample GSEA (ssGSEA) algorithm to quantify the infiltrating levels of 23 immune cells in the tumor microenvironment (TME)^33,34^. Since the Estimation of Stromal and Immune Cells in Malignant Tumors using Expression Data (ESTIMATE) algorithm^35^ could take advantage of the unique properties of the transcriptional profiles to infer the tumor cellularity as well as the tumor purity, we calculated immune and stromal scores for LUAD tumor samples using the ‘estimate’ R package. Tumor tissues with abundant infiltration of immune cells often has higher immune scores, with the opposite level of tumor purity. Moreover, we also applied another algorithm named ‘cell type identification by estimating relative subsets of RNA transcripts’ (CIBERSORT)^36^ (http://cibersort.stanford.edu/) to quantify the abundance of immune cell infiltration in the LUAD tumor samples. Then the samples with P-value less than 0.05 were used for further analysis.

### Estimation of drug sensitivity

IC50 (semi-inhibitory concentration) was an important indicator of assessing drug efficacy or response to sample treatment. Based on the sample transcriptome, we evaluated the IC50 for each sample using ‘pRRophetic’ R package^37^ to compare drug sensitivity across different IRGS subgroups. Higher IC50 represented lower drug sensitivity^38^. Moreover, we downloaded the files named ‘RNA: RNA-seq’ and ‘Compound activity: DTP NCI-60’ from the CellMiner (https://discover.nci.nih.gov/cellminer/home.do), further analyzed the correlation of FDA approved drugs Z scores with the IRG expression values.

### Additional bioinformatic and statistical analyses

The ‘limma’ R package was applied to performed the analysis of expression difference between LUAD tumor tissues and lung normal tissues, and the results were visualized by heatmaps. According to the data characteristics, Spearman or Pearson method were used for correlation analysis. Wilcoxon test and Kruskal–Wallis test were used to compare the statistical differences between two groups and multiple groups, respectively. The mutation landscape in different IRGS subgroups were presented using the ‘maftools’ R package. The Cox proportional hazards model was adopted to calculate the hazard ratios (HR) for each gene and clinical feature, through which the independent prognostic factors were ascertained simultaneously. We employed the ‘forestplot’ R package to visualize the results. Additionally, to assess the prognosis classification performance of the IRGS and other clinical factors, the ‘survivalROC’ R package was used to draw ROC curves, and the corresponding AUC were also estimated. Using the clinical characteristics and IRGS as input, multivariate Cox regression analysis with variable selection was implemented to identify the powerful combination of these predictors. Then, we built a quantitative nomogram with the ‘rms’ R package to predict the individual 1-, 3- and 5-year survival probabilities. To evaluate the prediction performance of the nomogram, the calibration curves, with the Hosmer–Lemeshow test, were used to judge the consistency between the model prediction values and the actual results. All statistical analyses were performed in R 3.6.2 software.

### Guidelines statement

This study obtained open data from the GEO database (https://www.ncbi.nlm.nih.gov/geo/) and TCGA (https://cancergenome.nih.gov/). All experimental protocols were performed in accordance with the relevant guidelines and regulations and adhered to the Declaration of Helsinki.

## Results

### The IRGS presents good evaluation performance in LUAD patients

**Figure 2:**
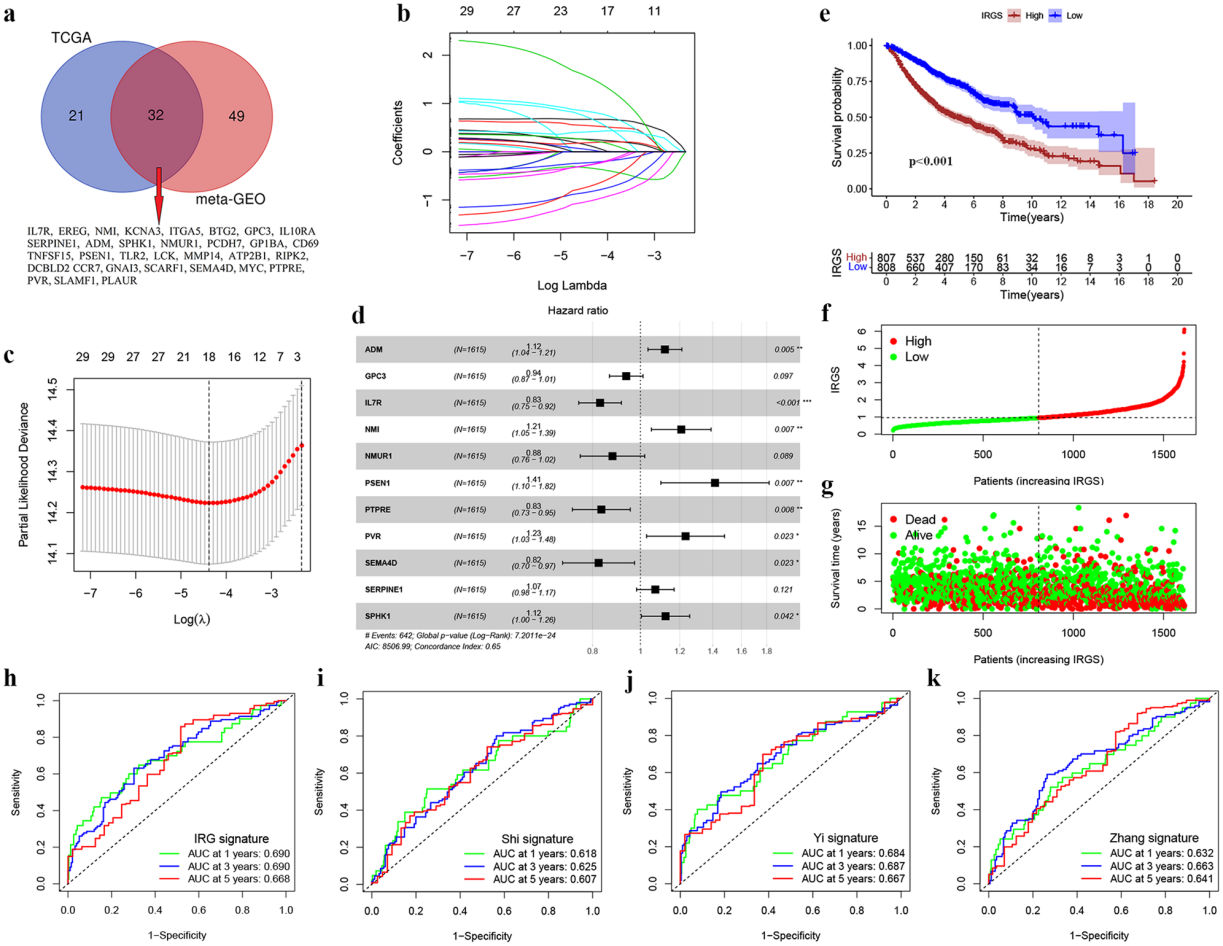
Controduction and verification of an IRG signature for LUAD patients. (**a**) Common genes of the prognostic-related genes in the meta-GEO and TCGA cohorts. (**b,c**) The Lasso regression analysis of the common genes to avoid the model overfitting. (**d**) Multivariate Cox analysis (stepwise regression) of the filtered genes in training dataset (meta-GEO cohort). (**e**) Kaplan–Meier survival curves revealed the OS differences between high- and low-IRGS groups in the meta-GEO cohort. (**f**) The risk score and (**g**) OS status distribution of the IRG signature in the meta-GEO cohort. (**h**) The ROC curve analysis of the IRG signature for predicting OS in the TCGA cohort. (**i**) The ROC curve analysis of the siganture constructed in the study of Shi et al. for predicting OS in the TCGA cohort. (**j**) The ROC curve analysis of the siganture constructed in the study of Yi et al. for predicting OS in the TCGA cohort. (**k**) The ROC curve analysis of the siganture constructed in the study of Zhang et al. for predicting OS in the TCGA cohort.

We obtained a total of 200 IRGs from the GSEA website, and their RNA expression profiles were applied for univariate Cox analysis. There were 81 prognosis-related IRGs (PRIRGs) in the meta-GEO cohort and 53 in the TCGA cohort (Fig. 2a, Table S3). The common PRIRGs in the two cohorts (n = 32) were conducted into LASSO analysis (Fig. 2b,c), and 18 genes (ADM, BTG2, GNAI3, GPC3, IL7R, ITGA5, MYC, NMI, NMURI, PCDH7, PLAUR, PSEN1, PTPRE, PVR, SEMA4D, SERPINE1, SPHK1, and TLR2) were performed in the multivariate Cox analysis (stepwise regression). Finally, a total of 11 genes (ADM, GPC3, IL7R, NMI, NMURI, PSEN1, PTPRE, PVR, SEMA4D, SERPINE1, SPHK1) were included in a predictive signature according to their risk coefficients (Fig. 2d, Table S4). We then evaluated the IRGS for each patient using the score calculation formula mentioned in the material section. With the median IRGS as cut-off, patients were classified into high- and low-IRGS groups. The Kaplan Meier survival curves of the two cohorts clearly showed that patients in low-IRGS group presented a longer overall survival (OS) than the high-IRGS patients (Fig. 2e, Fig. S1B). The distribution of patients with different IRGS and survival status were shown in Fig. 2f,g (the meta-GEO cohort, n = 1615) and Fig. S1C,D (the TCGA cohort, n = 500). As in Fig. 2h–k, the AUC values of IRGS constructed in this study (Fig. 2h) were significantly better than these signatures in other studies^28–30^ (Fig. 2i–k) in TCGA. The AUC values of ROC curves in the meta-GEO cohort at 1 year, 3 years and 5 years were 0.694, 0.678 and 0.658 respectively (Fig. S1A). Additionally, to verify the predictive performance of the IRGS in the different LUAD subgroups, subgroup analyses and the Kaplan–Meier survival curves were performed. As shown in Fig. S2, high-IRGS patients presented shorter OS than low-IRGS patients in most subgroups (≤ 65 years, > 65 years, female, male, stage I/II). In a large cohort of 1615 patients (the meta-GEO cohort), high-IRGS patients were also observed worse prognosis in stage III/IV subgroups (Fig. S2F). These above analyses indicated that the IRGS had good evaluation performance for risk stratification and prognosis prediction of LUAD patients.

### Multi-omics features of IRG signature genes reveal their great importance in LUAD

**Figure 3:**
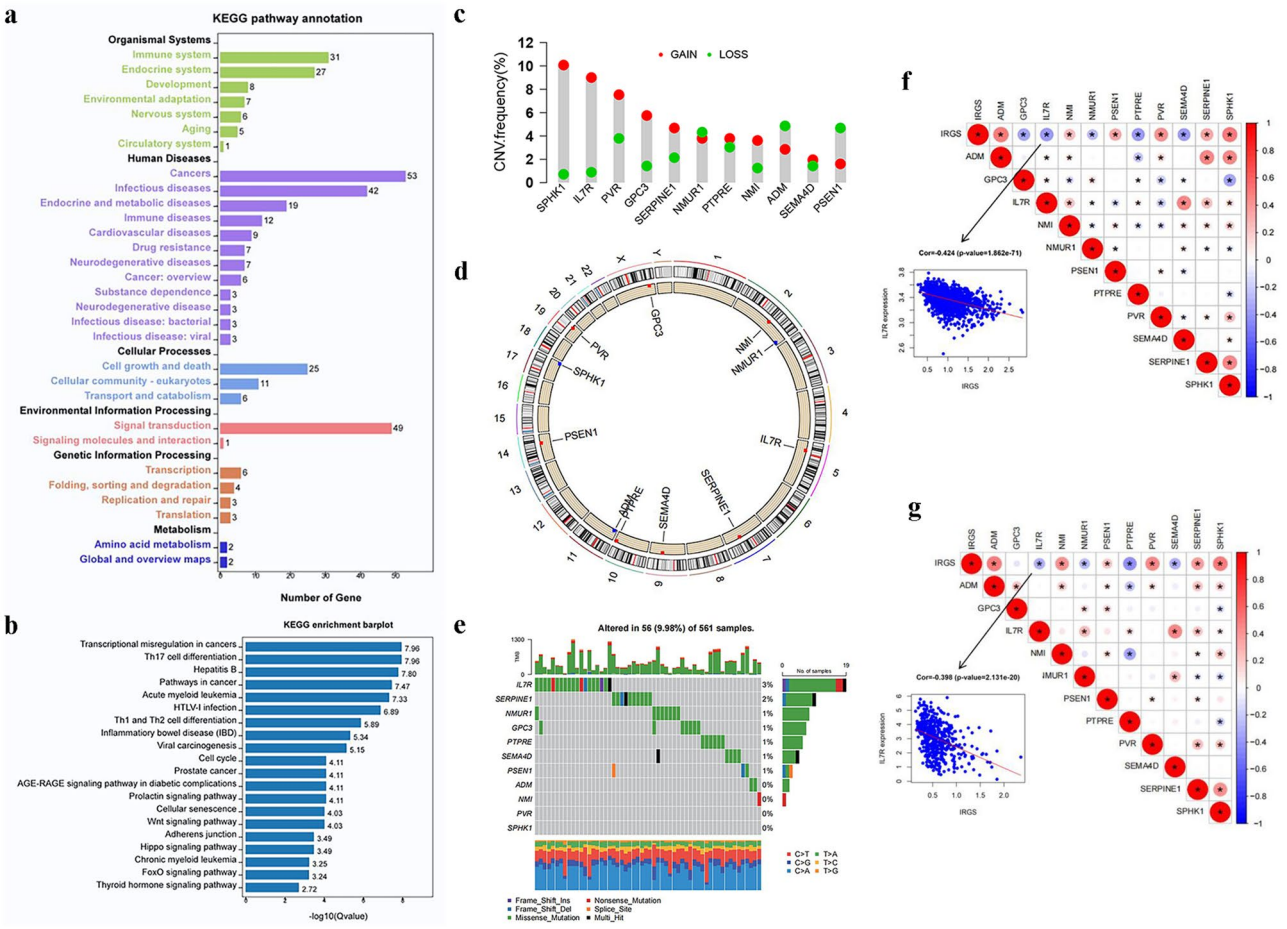
The landscape of genetic alterations of IRG signature genes in LUAD. (**a,b**) The KEGG enrichment analysis revealed the biological processes enriched for the IRG signature genes-related TFs. The KEGG database access link is as follows: https://www.kegg.jp/kegg/kegg1.html. (**c**) The CNV mutational frequency of the 11 IRG signature genes was prevalent. The deletion frequency, green dot; The amplification frequency, red dot. (**d**) The CNV alteration positions of these 11 genes on the chromosome. (**e**) 56 of the 561 LUAD patients experienced genetic alterations of the 11 genes, with a frequency of 9.98%, primarily including missense mutations, nonsense mutations and multi hit. The somatic mutation data were visualized using “maftool” R package. (**f,g**) Correlation analysis revealed the relationship between gene and gene, gene and IRGS in meta-GEO cohort (**f**) and TCGA cohort (**g**) (*p < 0.05; **p < 0.01; ***p < 0.001).

Based on the above analysis, we constructed an IRG signature containing 11 genes (ADM, GPC3, IL7R, NMI, NMURI, PSEN1, PTPRE, PVR, SEMA4D, SERPINE1, SPHK1). These genes were called as IRG signature genes. Most transcription factors (TFs) are associated with the cell cycle and play vital roles in the induction of proto-oncogene and tumor suppressor genes. We thus further explored the association of these 11 genes with 318 known TFs from the Cistrome program (http://cistrome.org/) (Table S5). By co-expression analysis, we finally identified 173 TFs associated with the 11 genes (Table S5). A functional analysis based on KEGG database^39^ indicated that these 173 TFs were involved in six major pathways (Fig. 3a). All the functional categories in these pathways were important, especially cancers, signal transduction, infectious diseases and immune system (Fig. 3a,b). Further analysis revealed that CNV of 11 IRG signature genes were prevalent. GPC3, IL7R, NMI, PTPRE, PVR, SEMA4D, SERPINE1 and SPHK1 showed widespread CNV amplification. In contrast, ADM, PSEN1 and NMURI had prevalent CNV deletions (Fig. 3c). And Fig. 3d visually showed the CNV alteration positions of these 11 genes on the chromosome. Innovatively, we further depicted the frequency of somatic mutations in these 11 genes in LUAD. In 561 samples, there was only 56 (9.98%) experienced genetic alterations, primarily including missense mutations, nonsense mutations and multi hit. Of these 11 genes, IL7R showed the highest mutation frequency, followed by SERPINE1 (Fig. 3e). We further explored the correlation between IRG signature gene expression and CNV as well as single nucleotide polymorphism (SNP) to clarify their potential relationship, and found that the CNV of some genes, such as PTPRE, PVR, IL7R, SEMA4D, NMI, PSEN1, significantly affected gene expression (Fig. S3A). Mutations in the IL7R gene weaken IL7R gene expression, while mutations in the other 10 genes did not seem to affect the expression levels of the corresponding genes (Fig. S3B). Subsequently, differentially expression analysis was performed, and it was found that many genes displayed different expression features in tumor tissues in different datasets (Fig. S4A–E). For example, IL7R was down-regulated in LUAD tumor tissues from GSE10072, TCGA and GSE40791, but up-regulated in GSE68465. Of these 11 IRG signature genes, only two genes (GPC3 and NMI) showed consistent changes in RNA expression. GPC3 was significantly down-regulated in tumor tissue in all data sets (Fig. S4G), while NMI was significantly up-regulated (Fig. S4F). We further validated the expression level of NMI and GPC3 in LUAD from the mRNA and protein levels. As shown in Fig. S5A–C, NMI was observably up-regulated in LUAD tumor tissue and cells (A549 and H1975). As expected, GPC3 was remarkably down-regulated in LUAD tumor tissue and cells (A549 and H1975) (Fig. S5D–F).

Correlation analysis revealed the relationship between genes and genes as well as genes and IRGS. In meta-GEO cohort, it was observed that IRGS was positively strongly related to the expression of ADM, NMI, PSEN1, PTPRE, PVR, SERPINE1 and SPHK1, and negatively correlated with GPC3, IL7R, NMURI, PTPRE and SEMA4D (Fig. 3f). The same findings were also observed in TCGA cohort (Fig. 3g). Also, we noted that the expression of IL7R and SEMA4D, and the expression of SERPINE1 and SPHK1 were significantly positively correlated in the two cohorts. Consistent with the results from univariate Cox analysis (Table S3), Kaplan–Meier survival analysis also revealed that the expression of ADM, GPC3, IL7R, NMI, NMURI, PSEN1, PTPRE, PVR, SEMA4D and SPHK1 significantly affected patient survival (Fig. S3C). Multivariate Cox analysis showed that ADM, IL7R, NMI, NMUR1, PTPRE, PVR, SEMA4D and SPHK1 were independent prognostic factors of LUAD, where ADM, NMI, PTPRE, PVR, SPHK1 were prognostic risk factors, while IL7R, NMUR1 and SEMA4D were favorable prognostic factors (Fig. 2d, Table S4).

The analyses mentioned above told about the multi-omics traits of the 11 IRG signature genes in LUAD. The effect on prognosis, transcriptomic (abnormal expression of GPC3 and NMI) and genomic (mutations of IL7R, CNV of PTPRE, PVR, IL7R, SEMA4D, NMI and PSEN1) alterations suggested that these IRG signature genes were of great importance in LUAD.

### Comprehensive analyses reveal different biological processes and TME between two IRGS subgroups

**Figure 4:**
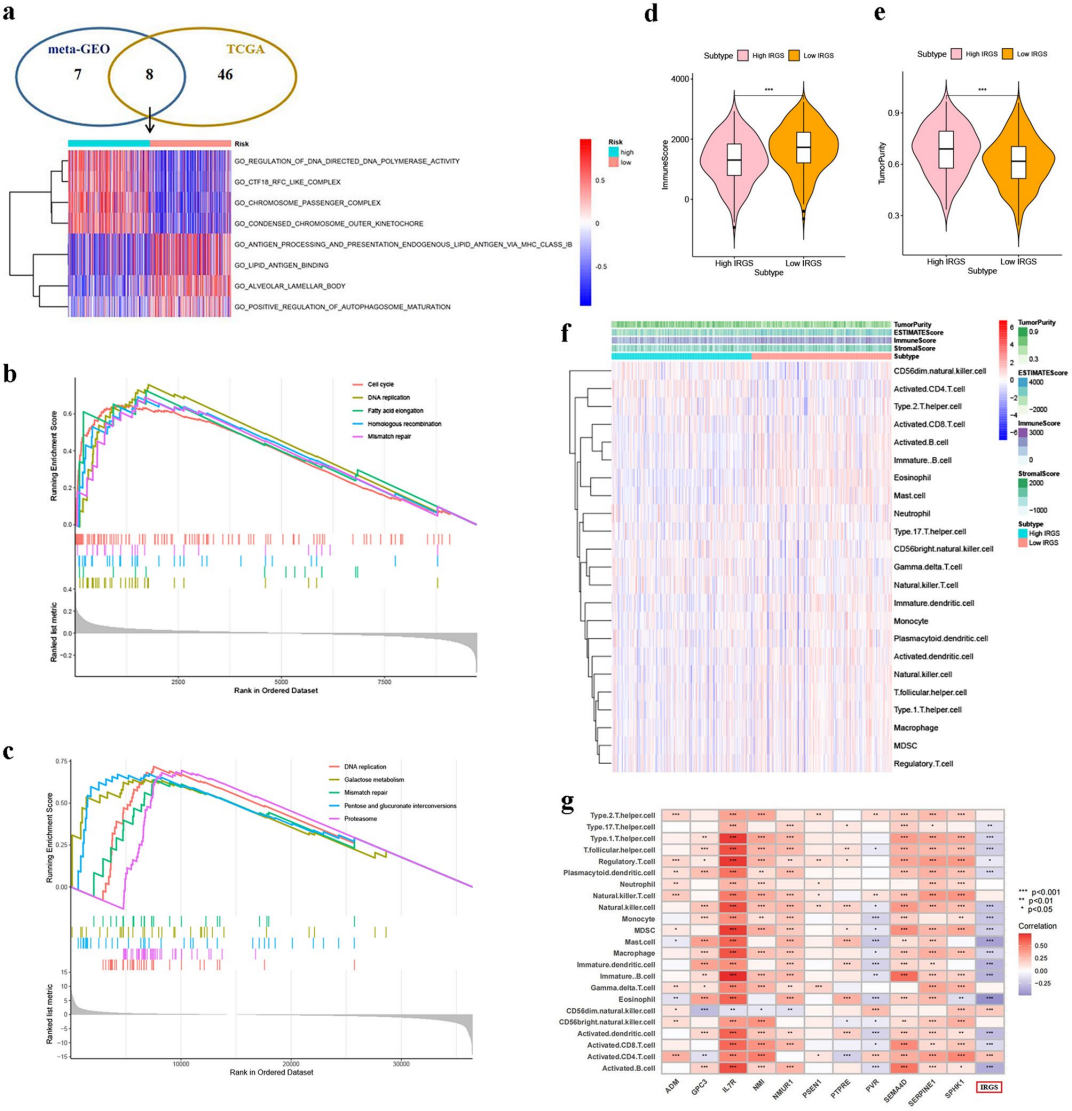
Enriched biological pathways and tumor immune microenvironment characteristics in different IRGS subgroups. (**a**) The GSVA revealed eight co-enriched pathways obtained from both cohorts (meta-GEO and TCGA cohorts). The heatmap was applied to visualize the enriched biological pathways, and red indicated the activated pathways and blue indicated the inhibited pathways. (**b**) GSEA enrichment analysis based on KEGG in meta-GEO cohort. (**c**) GSEA enrichment analysis based on KEGG in TCGA cohort. (**d,e**) Comparison of immune score and tumor purity in the high- and low-IRGS groups. (**f**) Comparison of tumor immune infiltrating cells based on ssGSEA algorithm in the high- and low-IRGS groups. Red indicated the high infiltrating levels of immune cells and blue indicated the low infiltrating levels. ESTIMATE score, stroma score, immune score, tumor purity and subtype are shown in annotations above. (**g**) Correlation between the abundance of tumor immune cells infiltrating using ssGSEA algorithm and IRG signature gene expression as well as IRGS (*p < 0.05; **p < 0.01; ***p < 0.001).

To infer the biological behaviors between different IRGS subgroups, GSVA and GSEA analyses were performed. The GSVA revealed eight co-enriched pathways obtained from both cohorts (meta-GEO and TCGA cohorts) (Fig. 4a, Table S6), where regulation of DNA directed DNA polymerase activity, Ctf18 RFC-like complex, chromosome passenger complex and condensed chromosome outer kinetochore were enriched in the high-IRGS group, while antigen processing and presentation endogenous lipid antigen via MHC class IB, lipid antigen binding, alveolar lamellar body, and positive regulation of autophagosome maturation were enriched in the low-IRGS group. The GSEA also revealed that there were two co-enriched pathways among the top five pathways obtained from both cohorts. These two pathways including DNA replication and mismatch repair were enriched in the high-IRGS group (Fig. 4b,c, Table S7). Additionally, ESTIMATE algorithm was used to quantify the immune score and tumor purity for each sample. The wilcoxon rank sum test revealed significant differences in immune score and tumor purity between low- and high-IRGS groups. These results from ESTIMATE algorithm told about that the high-IRGS patients presented higher tumor purity, and the opposite was true for immune scores (Fig. 4d,e). Immune-infiltrating cells played important roles in TME, and we subsequently quantified immune-infiltrating cells in each IRGS subgroup using ssGSEA and CIBERSORT algorithms to further explain the potential association between risk stratification and TME in LUAD. ssGSEA revealed the low IRGS tumors were significantly infiltrated by activated B cell, immature B cell, activated CD8 T cell, immature dendritic cell (DC), activated DC, plasmacytoid DC, Type 1T helper cell, T follicular helper cell, Type 17T helper cell, NK cell, NK T cell, eosinophil, macrophage, MDSC and mast cell. While activated CD4 T cell, CD56dim NK cell significantly infiltrated in the high IRGS tumors (Fig. 4f, Fig. S6A). CIBERSORT also showed that significant differences in immune cells infiltrating level were observed between high and low IRGS tumors (Fig. S6B). The results from ssGSEA and CIBERSORT might help to explain why the low-IRGS group presented higher immune scores and lower tumor purity. We also compared the abundance of immune infiltrating cells between LUAD normal and tumor tissues (Fig. S6C), and further explored the correlation between the abundance of different immune infiltrating cells. It was found that LUAD tumor tissue was significantly infiltrated by B cells naive, macrophages M1, plasma cells, DCs resting, T cells CD4 memory activated, T cells follicular helper and Tregs. While T cells CD4 memory resting, NK cells resting, mast cells resting, monocytes, neutrophils, macrophages M0/2, eosinophils significantly infiltrated in LUAD normal tissue. As shown in Fig. S6D, mast cells resting showed a significantly positive correlation with NK cells activated (Cor = 0.35), and plasma cells showed a significantly negative correlation with macrophages M2 (Cor =  − 0.45). Given the differential infiltrating abundance of immune cells in different tissues (high, low IRGS tumor and normal tissues), we investigated the relationship between the immune cell infiltrating level and patient prognosis. The results showed that except T cells gamma delta, other cells did not affect the survival of patients (Fig. S7). However, between the different tissues, we did not observe the difference in the T cells gamma delta infiltrating level (Fig. S6B,C). Further investigation in terms of the relationship between TME infiltrating cells and IRG signature genes as well as IRGS, we found that IRG signature genes and IRGS were positively or negatively strongly related to at least 7 cell types based on the ssGSEA method, in which IL7R was significantly positively related to a large number of immune cells, while IRGS were significantly negatively correlated with most immune cells (Fig. 4g). This result varied somewhat from the results based on the CIBERSORT algorithm (Fig. S6E), probably due to the internality of the different algorithms and the immune cell abundance calculated by CIBERSORT method with more emphasis on the cell status and subtypes. This also further illustrated the complexity of TME. Taken together, the high- and low-IRGS groups based on IRG signature had significantly different enrichment pathways and TME. We speculated that the abnormal expression of IRG signature genes, the remodeling of TME and the change of tumor biological pathways affected each other, resulting in different prognosis of patients.

### The potential of IRGS in predicting therapeutic responses

**Figure 5:**
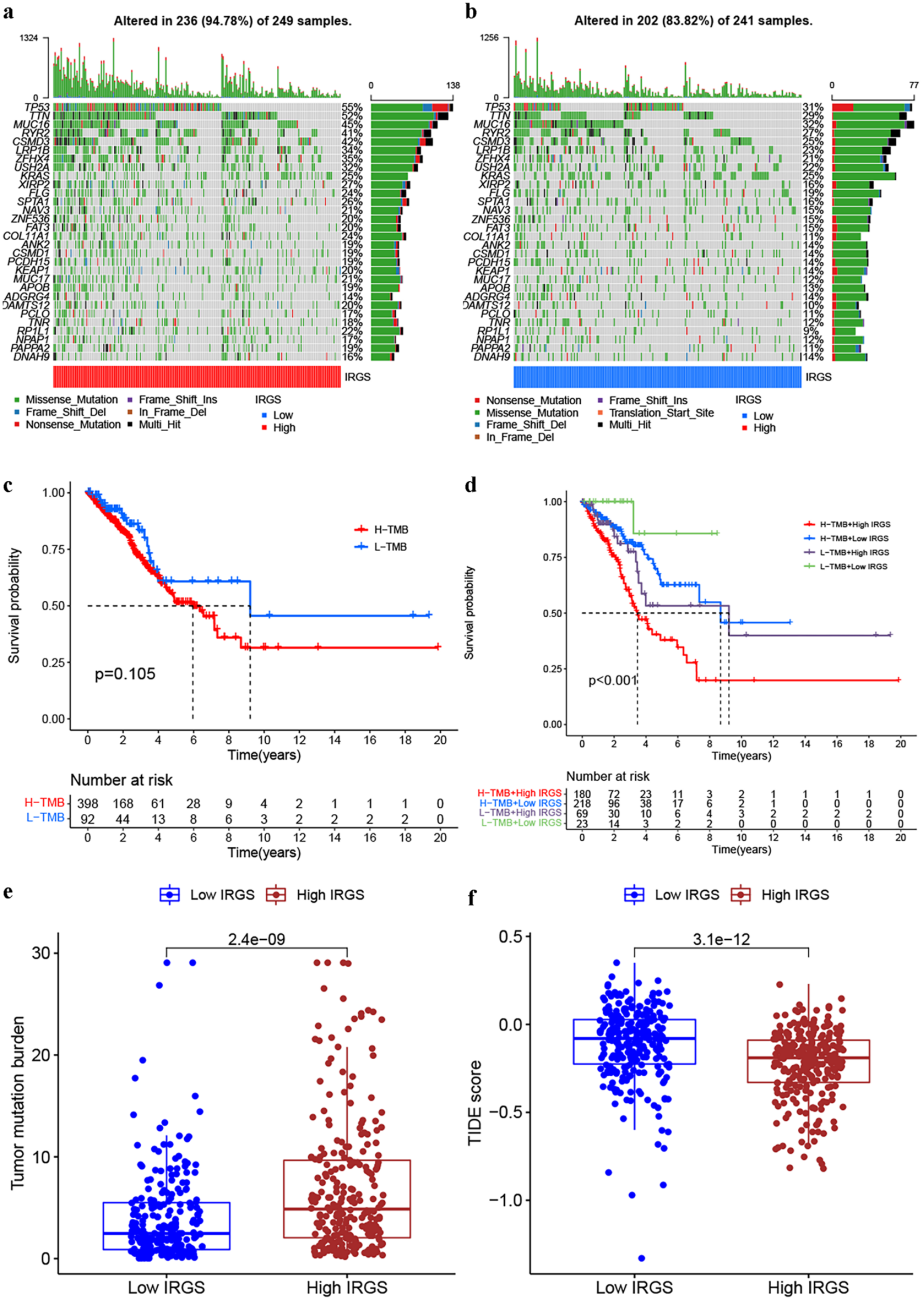
Comparison of the genetic characteristics between high- and low-IRGS groups and prediction of immune efficacy. (**a,b**) Somatic mutation characteristics in the high (**a**) and low (**b**) IRGS groups based on the TCGA cohort. (**c**) The Kaplan–Meier survival curves revealed the survival differences in patients with high and low TMB. (**d**) The cross-talk among TMB, IRGS and patient survival. (**e**) The TMB status of the high- and low-IRGS groups were analyzed and plotted. (**f**) The TIDE scores of the high- and low-IRGS groups were analyzed and plotted.

To reveal the somatic mutational landscapes for insight into the mutational process of LUAD, we made a genome-wide somatic mutation profile comparison between different risk subgroups based on TCGA data. Figure 5a,b displayed the mutation landscapes of the top 30 most common mutant genes in the high- and low-IRGS groups of LUAD, respectively. TP53, TTN, and MUC16 for LUAD, were the top three most frequently mutated genes identified in this study. The high-IRGS group exhibited a significantly higher proportion of specific gene mutations. This enabled us to more comprehensively describe the impact of IRGS risk stratification on genomic alterations. Additionally, the Kaplan–Meier survival analysis was performed to further understand the cross-talk among somatic mutations, IRGS and patient survival. This results revealed that tumor mutation burden (TMB) had little to do with patient prognosis (Fig. 5c), but patients with high TMB combined with high IRGS presented the worst prognosis. In contrast, patients with low TMB combined with low IRGS predicted best (Fig. 5d). Increasing evidence^40,41^ has demonstrated the association between TMB and immunotherapy response. Hence, we compared the distribution of TMB in the high-and low-IRGS groups and found higher TMB in the high-IRGS group (Fig. 5e), which was consist with the results from Fig. 5a,b that high-IRGS group exhibited significantly higher mutation frequencies. The above analyses initially indicated that high-IRGS patients may present a higher response rate to immunotherapy. To further confirm this conclusion, we applied a website named Tumor Immune Dysfunction and Exclusion (TIDE) (http://tide.dfci.harvard.edu) to compute TIDE scores for different IRGS subgroups. TIDE scores could be calculated for each sample by TIDE website based on the transcriptome data^42^. A lower TIDE score showed a higher response rate against both PD-1 and anti-CTLA-4 drugs. Our analysis revealed that the TIDE score was remarkably increased in the low-IRGS group (Fig. 5f). To sum up, patients at high IRGS may have a better immune response when receiving immune checkpoints inhibitors.

**Figure 6:**
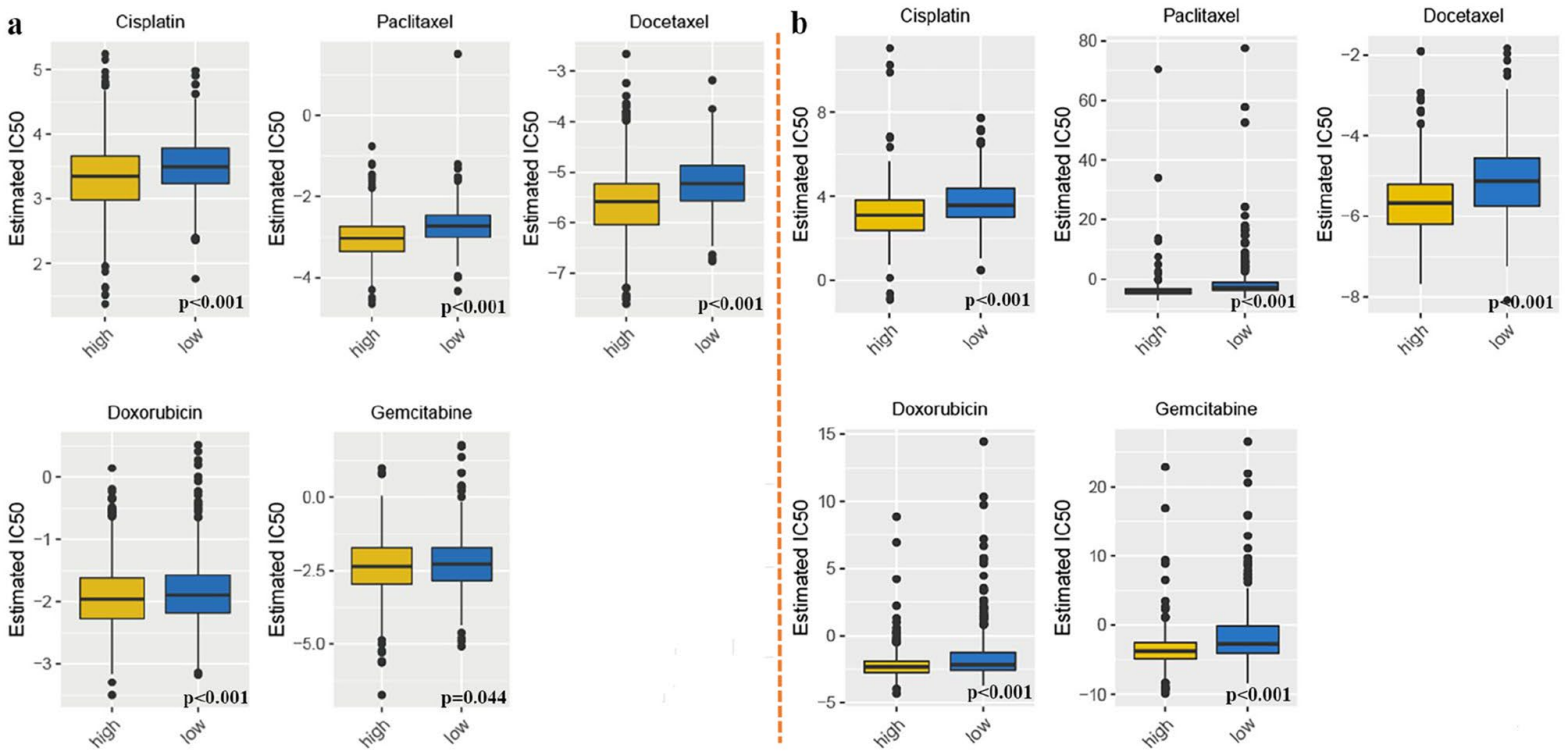
Drug sensitivities comparison between different IRGS subgroups. The estimated IC50s of cisplatin, paclitaxel, docetaxel, doxorubicin, and gemcitabine in the high- and low-IRGS groups in the meta-GEO cohort (**a**) and TCGA cohort (**b**).

Chemotherapy has been widely used in the treatment of many malignancies, including LUAD. Not all patients clinically benefited from these treatments, so it was of great importance to identify subgroups of patients who may be more sensitive to certain drugs. Based on the sample transcriptome, we here evaluated and compared drug sensitivity across different IRGS subgroups. As shown in Fig. 6a,b, the IC50s of several commonly-used drugs (cisplatin, paclitaxel, docetaxel, doxorubicin, gemcitabine) were lower in the high-IRGS group, indicating that high-IRGS patients might show higher sensitivity to these drugs.

Additionally, CellMiner analysis also revealed the correlation of the 11 IRG signature genes and FDA approved drugs. Fig. S8A–P showed the correlation between the first 16 drugs (p values small to large) and IRG signature genes. Some of these genes (such as PSEN1, PTPRE, SPHK1, PVR, SERPINE1, NMUR1) showed the correlation with the predicted drugs (Table S8). The correlation network map was visualized in Fig. S9. The above results preliminarily revealed the relationship between risk stratification as well as IRG signature gene expression and the sensitivity of commonly-used drugs, which could provide valuable clues to the development of individualized therapeutic strategies for LUAD patients.

### Establishment of a prognostic nomogram to optimize survival prediction in LUAD patients

**Figure 7:**
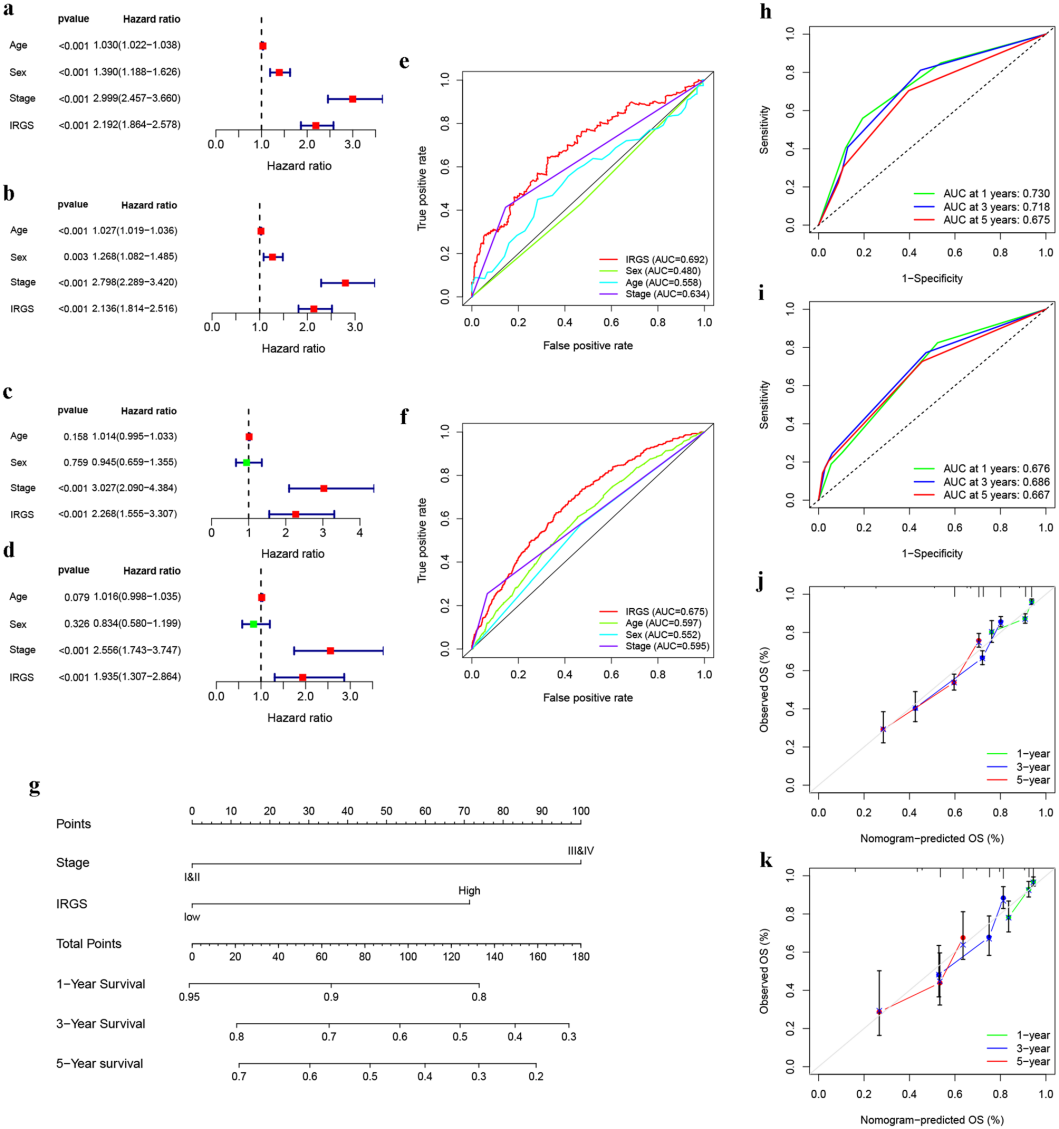
Construction and validation of a nomogram to optimize survival prediction. (**a–d**) IRGS was an independent prognostic predictor by univariate and multivariate Cox analyses (**a**) univariate Cox analysis in meta-GEO cohort; (**b**) multivariate Cox analysis in meta-GEO cohort; (**c**) univariate Cox analysis in TCGA cohort; (**d**) multivariate Cox analysis in TCGA cohort.). (**e,f**) ROC curves for age, gender, stage and IRGS in meta-GEO cohort (**e**) and TCGA cohort (**f**). (**g**) Nomogram based on IRGS and TNM stage. (**h,i**) ROC curves for the nomogram in meta-GEO cohort (**h**) and TCGA cohort (**i**). (**j,k**) Calibration curves of the nomogram for predicting 1, 3 and 5-year OS probability in meta-GEO cohort (**j**) and TCGA cohort (**k**).

Independent prognostic analyses were respectively performed based on data from the meta-GEO cohort of 1615 patients and the TCGA cohort of 490 patients containing detailed clinical information. As shown in Fig. 7a–d, in both cohorts, clinical stage and IRGS were considered as independent prognostic factors for LUAD patients. To assess the estimated performance of IRGS in predicting the prognosis of patients, we further calculated the AUC values, and found that 3-year AUC values of the IRGS in two cohorts were 0.675 and 0.692, respectively, both higher than other clinicopathologic factors (such as age, sex and stage) (Fig. 7e,f). Based on the two independent prognostic factors, we established a prognostic nomogram for LUAD patient in the meta-GEO cohort (Fig. 7g). The ROC analyses indicted the nomogram presented a powerful capacity for survival prediction with high AUC values in both the meta-GEO and TCGA cohorts (Fig. 7h,i). Moreover, the calibration curves in both cohorts also presented favourable consistency with the ideal performance (Fig. 7j,k), indicating a high accuracy of the nomogram.

## Discussion

In view of the complex oncogenic mechanisms and tumor heterogeneity in LUAD, the development of personalized management strategies and accurate prediction of patient prognosis remain extremely challenging. IRGs play crucial roles in tumorigesis and progression^14,15^, but the relationship between IRGs and LUAD prognosis as well as the response to drug therapy remains poorly investigated. Therefore, based on IRG expression profiles, this study attempts to develop a model for prognosis risk stratification and drug therapy response prediction in LUAD patients, hoping to provide reference for clinical decision-making and future studies. In the present study, based on as many as possible LUAD patients (n = 1615) collected from multiple GEO chips, we developed a prognostic IRG signature that could effectively identify high- and low-risk patients. Subsequently, prognostic power was validated in an independent TCGA cohort of 500 patients. Notably, in the meta-GEO and TCGA cohorts, we observed that the IRGS, as with TNM stage, was an independent prognostic factor for LUAD patients, and its prognostic capacity was also superior to some clinicopathologic parameters (e.g., TNM stage, age, gender). In the meta-GEO cohort, the 3-year and 5-year AUC values of the model constructed in this study were not as good as those of Yi et al.^28^, but this result was still acceptable and interpretable. Compared with the models constructed in previous studies^28–30^, the IRG signature constructed in this study still exhibited ideal predictive performance, which was a parallel comparison based on TCGA data sources. Furthermore, in some LUAD patient subgroups, the IRG signature retained its predictive power to effectively distinguish between high- and low-IRG patients.

A growing number of studies have proved that tumorigenesis and progression were closely related to TME^43–46^, where various cytokines, chemokines and cells interacting with tumor cells, especially immune cells, were increasingly considered to play key roles in tumor resistance in vivo. In this study, multiple methods (CIBERSORT, ssGSEA and ESTIMATE) were used to depict the TME landscapes of different IRGS subgroups. We observed that low-IRGS group had more types of immune cell infiltrating, with anti-tumor cells (such as CD8^+^ T cells, macrophage, NK cells, dendritic cells and so on) as well as immunosuppressive cells (e.g., MDSC). Tumor tissues infiltrated by abundant immune cells had higher immune score and lower tumor purity. And the results from ESTIMATE algorithm (the low-IRGS group had higher immune score and lower tumor purity) also further confirmed the above-stated conclusion. Previous studies^47–51^ have revealed the relationship between tumor purity as well as immune score and patient survival. In TME, the percentage of tumor cells was called tumor purity. It has been reported that the poor prognosis of glioma^51^ and colorectal cancer^47^ was closely associated with low tumor purity. In contrast to the above results, Wang et al.^50^ and our previous study^49^ observed that low tumor purity tended to suggest a better prognosis in LUAD patients. Our finding in this study was also in line with that of the above-stated studies. Tumor purity of different cancer patients presented sharply different indicative significance, seemingly highlighting the potential differences in the pattern of tumorigenesis and progression of different tumors. It is clear that tumor tissue contains not only tumor cells, but also non-tumor cells, such as stromal cells and immune cells. These non-tumor cells dilute the purity of the tumor and play an important role in the biological process of the tumor. As described by Zhang et al.^51^, the purity of gliomas was associated with distinct patterns of genomic alterations. This intrinsic driving force contributed to differences in phenotype and survival among patients with different tumor purities of the same cancer type. It is well known that different cancers have significant heterogeneity, including genomic heterogeneity. And it is the existence of this heterogeneity that should be responsible for the suggestive significance of tumor purity in different cancers. In addition, an extremely important factor that should not be ignored is the local immune status of the tumor. Lower purity means stronger local immune status^51^, which is closely related to the composition, proportion and activation status of different immune cells. This further illustrates the remarkable complexity of the tumor microenvironment, and further research is needed to unravel its mysteries.

Additionally, enriched biological processes and genomic alterations were depicted in different IRGS subgroups. Some critical cellular pathways indicating vital processes, such as regulation of DNA directed DNA polymerase activity, DNA replication and mismatch repair were enriched in the high-IRGS group. This might help explain why high-IRGS group presented more aggressive molecular changes than the low-IRGS group. Correlated with the differences of genomic alterations in different IRGS subgroups, it was not astonished to find that high-IRGS group had higher TMB, but further analysis revealed no dramatic correlation between patient prognosis and high/low TMB. However, the cross-talk between TMB, IRGS and patient prognosis revealed that patients with high TMB combined with high IRGS embraced the worst prognosis, and patients with low TMB combined with low IRGS had the best prognosis. This forwardly confirmed the predictive efficacy of IRGS in risk stratification, and also highlighted the complexity of the potential link between tumor genomic alterations and prognosis.

The best strategy for personalized immunotherapy should be to seek effective biomarkers to predict sensitivity to drug therapy. Reliable biomarkers have not been met in clinical practice. Accumulated evidence^40,41^ has demonstrated patients with high TMB status presented durable clinical responses to immunotherapy. In our study, the high-IRGS group presented higher TMB, and TIDE score was remarkably increased in the low-IRGS group. Additionally, chemotherapy has been widely used in the treatment of many malignancies, including LUAD. Here, we predicted the sensitivity of different IRGS subgroups to several commonly-used drugs. We found the IC50s of several commonly-used drugs were lower in the high-IRGS group. Thus, the above results indicated that high-IRGS patients might benefit more from immunotherapy and commonly prescribed agents (cisplatin, paclitaxel, docetaxel, doxorubicin, gemcitabine), and fully demonstrated the values of IRGS in predicting drug therapeutic responses. To sum up, these results might provide additional clues for individualized treatment for LUAD patients. Additionally, our study also explored the correlation between IRG signature gene expression and drug Z-scores, which would provide directional suggestions and preliminary basis for drug development of gene-targeted therapy in human cancer.

In recent years, nomogram has been widly used in prognostic assessment of cancer patients^52,53^. To further optimize the survival prediction of LUAD patients, we established a prognostic nomogram including TNM stage and IRGS. Importantly, the nomogram demonstrated reliable accuracy and robustness in predicting survival for LUAD. It could therefore help clinicians accurately determine the prognosis of patients and develop individualized treatment regimens.

There remained some limitations in this study, and the nature of retrospective research was an inevitable question. While as many data sets as possible were included for rigorous model building and validation, as well as ‘Combat’ approach to reduce batch effects, we were still unable to fully resolve the sampling deviation caused by cross-platform integration. In addition, although this study used a large number of retrospective data sets for risk stratification and efficacy prediction, there was still a lack of appropriate LUAD data sets based on immunotherapy regimen to verify the predictive robustness of the IRGS, so as to further strengthen our conclusion.

Overall, the present study identified and validated a novel IRG signature for risk stratification and efficacy prediction in over 2000 LUAD samples, described the multi-dimensional characterization of 11 IRG signature genes in LUAD, and emphasized the essential roles of IRG signature genes in shaping the complexity of TME in LUAD. More broadly, assessing the IRGS of LUAD would help to enhance our perception of immune cell infiltrating characteristics and provide important insights into the efficacy of drug therapy (immunotherapy, and chemotherapy). To make a long story short, our study might inform important treatment strategies, finally promoting the individualized management of LUAD patients.

## Supplementary Information

Supplementary Figures.

Supplementary Table S1.

Supplementary Table S2.

Supplementary Table S3.

Supplementary Table S4.

Supplementary Table S5.

Supplementary Table S6.

Supplementary Table S7.

Supplementary Table S8.

## Data Availability

This study obtained open data from the GEO database (https://www.ncbi.nlm.nih.gov/geo/) and TCGA (https://cancergenome.nih.gov/). The data sets generated and analyzed during the present study are available from the corresponding author on reasonable request.

## Abbreviations

LUAD: Lung adenocarcinoma
NSCLC: Non-small cell lung cancer
TME: Tumor microenvironment
IRGs: Inflammatory response genes
IRGS: Inflammatory response gene scores
TCGA: The Cancer Genome Atlas
GEO: Gene Expression Omnibus
GSVA: Gene set variation analysis
GSEA: Gene-set enrichment analysis
CNV: Copy number variation
DEGs: Differentially expressed genes
ROC: Receiver operating characteristic curve
AUC: Area under the curve
ssGSEA: Single sample GSEA
ESTIMATE: The estimation of stromal and immune cells in malignant tumors using expression data
CIBERSORT: Cell type identification by estimating relative subsets of RNA transcripts
IC50: Semi-inhibitory concentration
TIDE: Tumor immune dysfunction and exclusion
TMB: Tumor mutation burden
