# Supplementary material for: Identification of an inflammatory response signature associated with prognostic stratification and drug sensitivity in lung adenocarcinoma
Source: Sci Rep. 2022 Jun 16;12:10110. doi: 10.1038/s41598-022-14323-6 (PMC9203558; doi:10.1038/s41598-022-14323-6)
Supplement: Supplementary file 1 — Supplementary Figures. [file 41598_2022_14323_MOESM1_ESM.docx]

**Identification of an inflammatory response signature associated with prognostic stratification and drug sensitivity in lung adenocarcinoma**

Congkuan Song **^1#^**, Zilong Lu **^1#^**, Kai Lai **^1#^**, Donghang Li ^1^, Bo Hao ^1^, Chenzhen Xu ^1^, Shize Pan ^1^, Ning Li ^1^ and Qing Geng ^1*^

1. Department of Thoracic Surgery, Renmin Hospital of Wuhan University, Wuhan, China.

**Supplemantary Figures:**

**
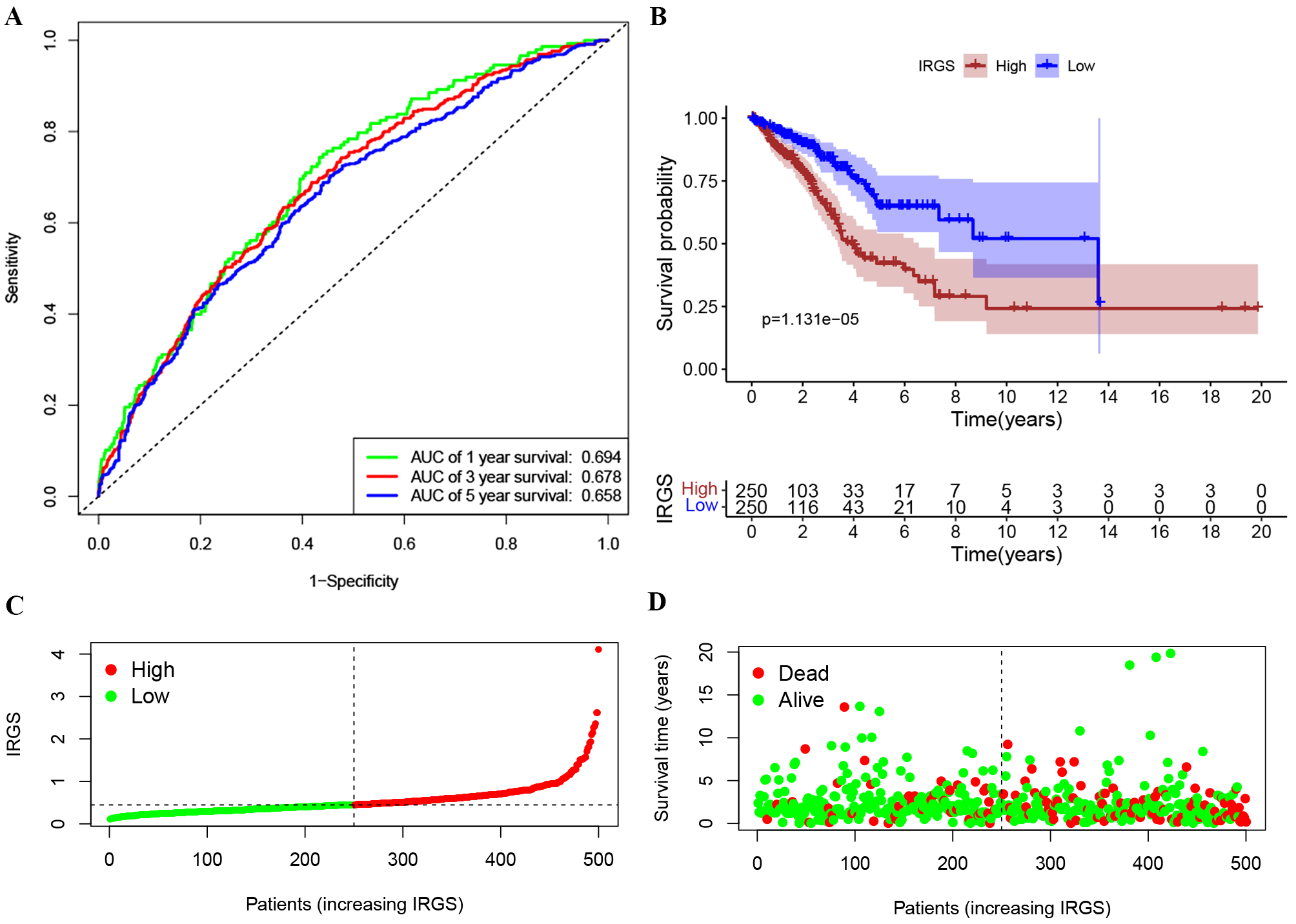
**

**Figure S1. Evaluation of the IRG signature to predict OS.** (**A**) The ROC curve analysis of the signature for predicting OS in the meta-GEO cohort. (**B**) Kaplan-Meier survival curves for OS based on the IRGS in the TCGA cohort. The tick-marks on the curve represent the censored subjects. The number of patients at risk is listed below the curve. The IRGS (**C**) and OS status (**D**) distribution of the signature in the TCGA cohort.


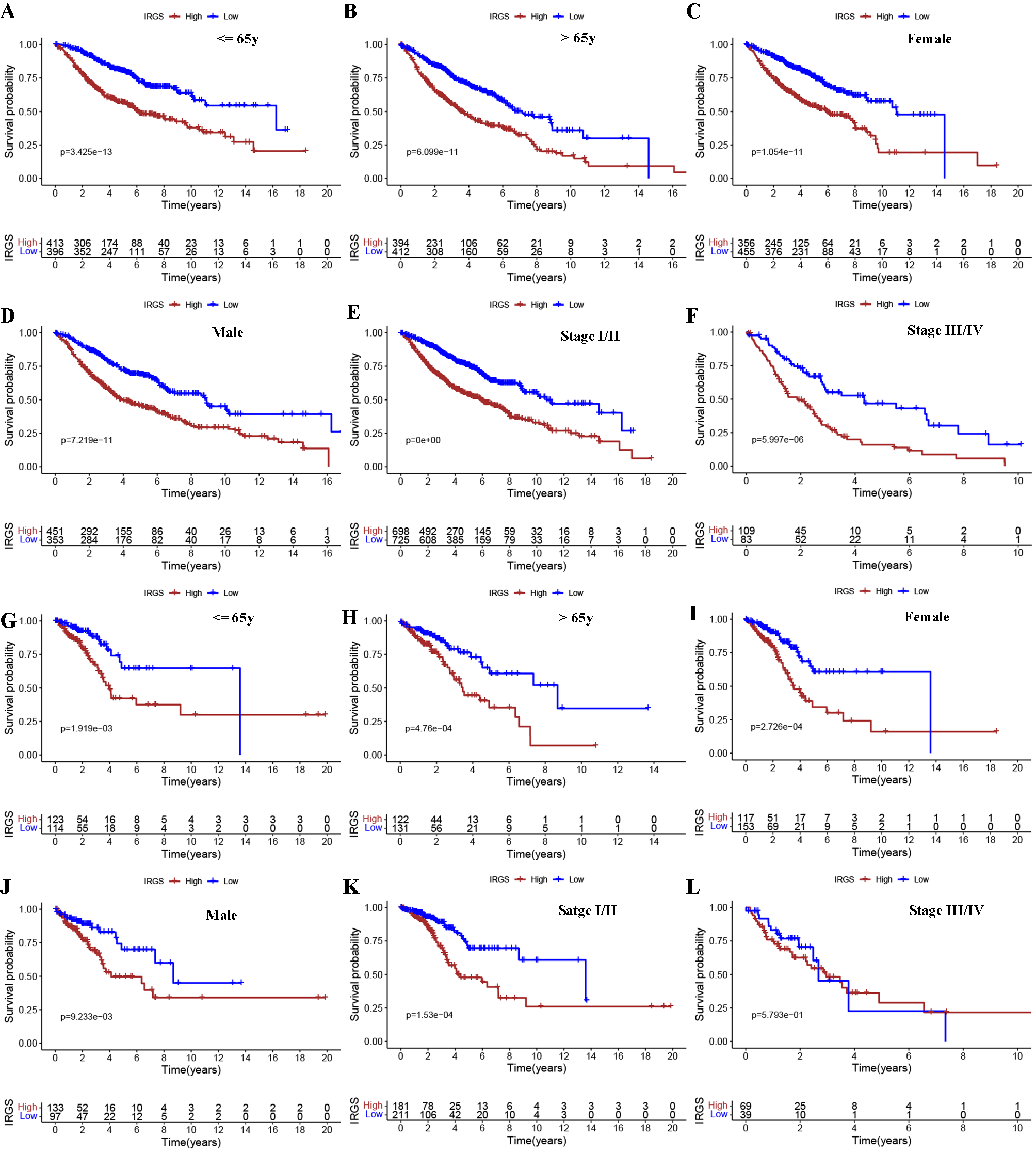


**Figure S2. The subgroup analyses to verify the predictive performance of IRGS in the different LUAD subgroups.** Kaplan-Meier survival curves were plotted in different subgroups in meta-GEO cohort (**A-F**) and TCGA cohort (**G-L**).

**
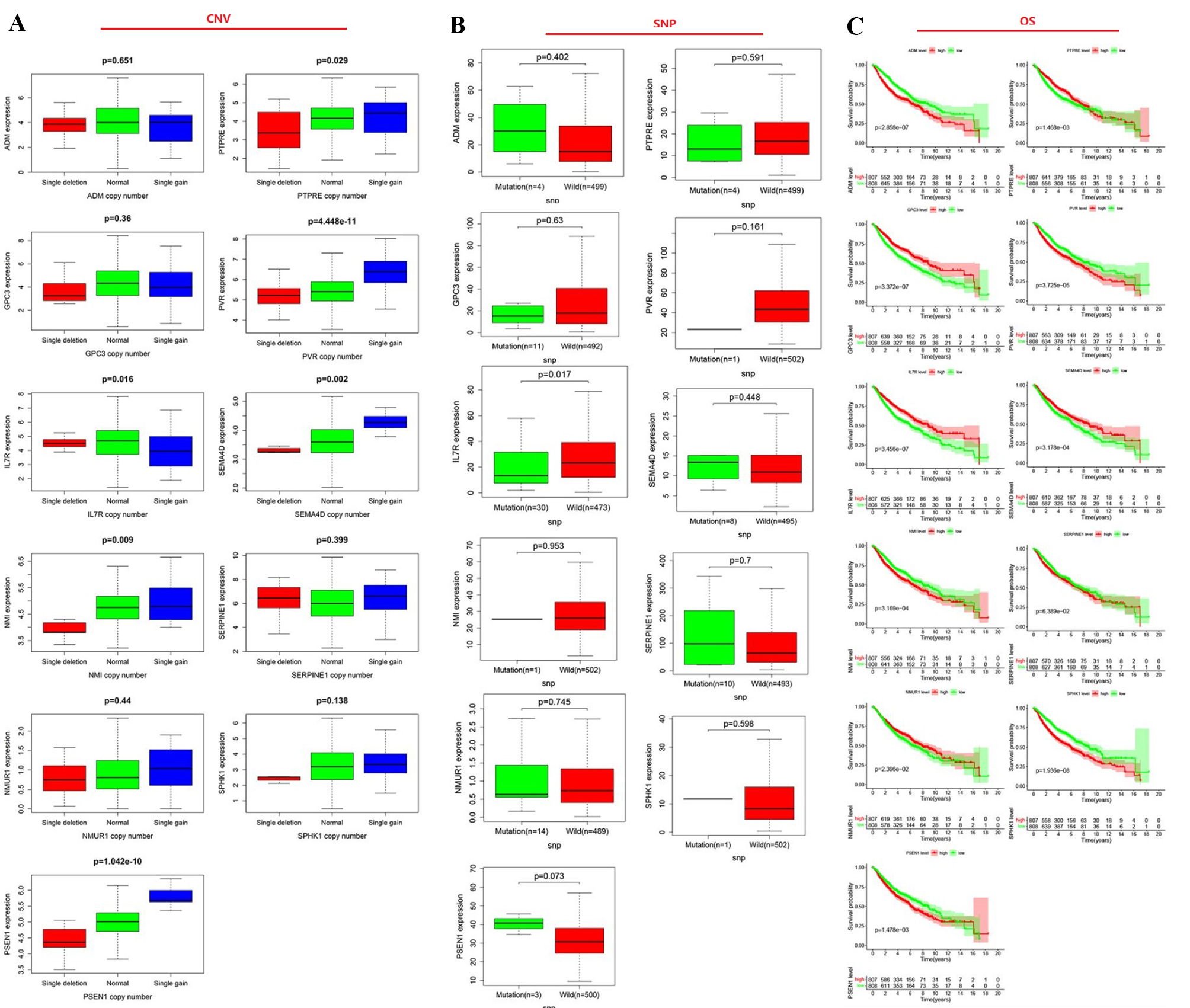
**

**Figure S3. Correlation of IRG signatture gene expression with CNV, SNP and OS.** (**A**) Relationship between gene expression and CNV based on the TCGA cohort. (**B**) Relationship between gene expression and SNP based on the TCGA cohort. （**C**）Relationship between gene expression and OS based on the meta-GEO cohort.


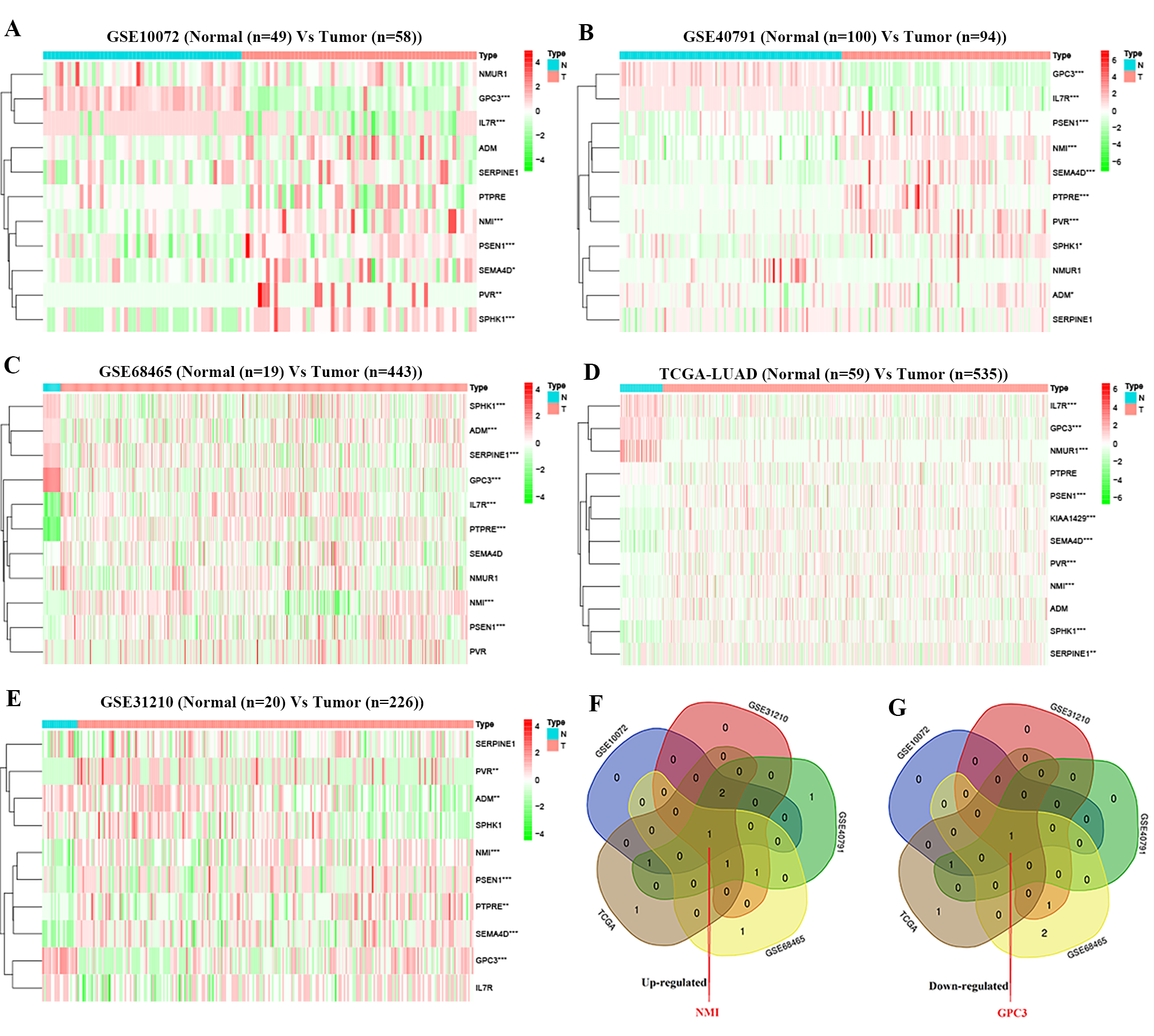


**Figure S4. Different expression features of IRG signature genes in LUAD tumor tissues from different datasets. (A)** GSE10072. (**B**) GSE40791. (**C**) GSE68465. (**D**) TCGA-LUAD. (**E**) GSE31210. (**F**) Venn diagram, NMI was up-regulated in LUAD tumor tissues in all datasets. (**G**) Venn diagram, GPC3 was down-regulated in LUAD tumor tissues in in all datasets.


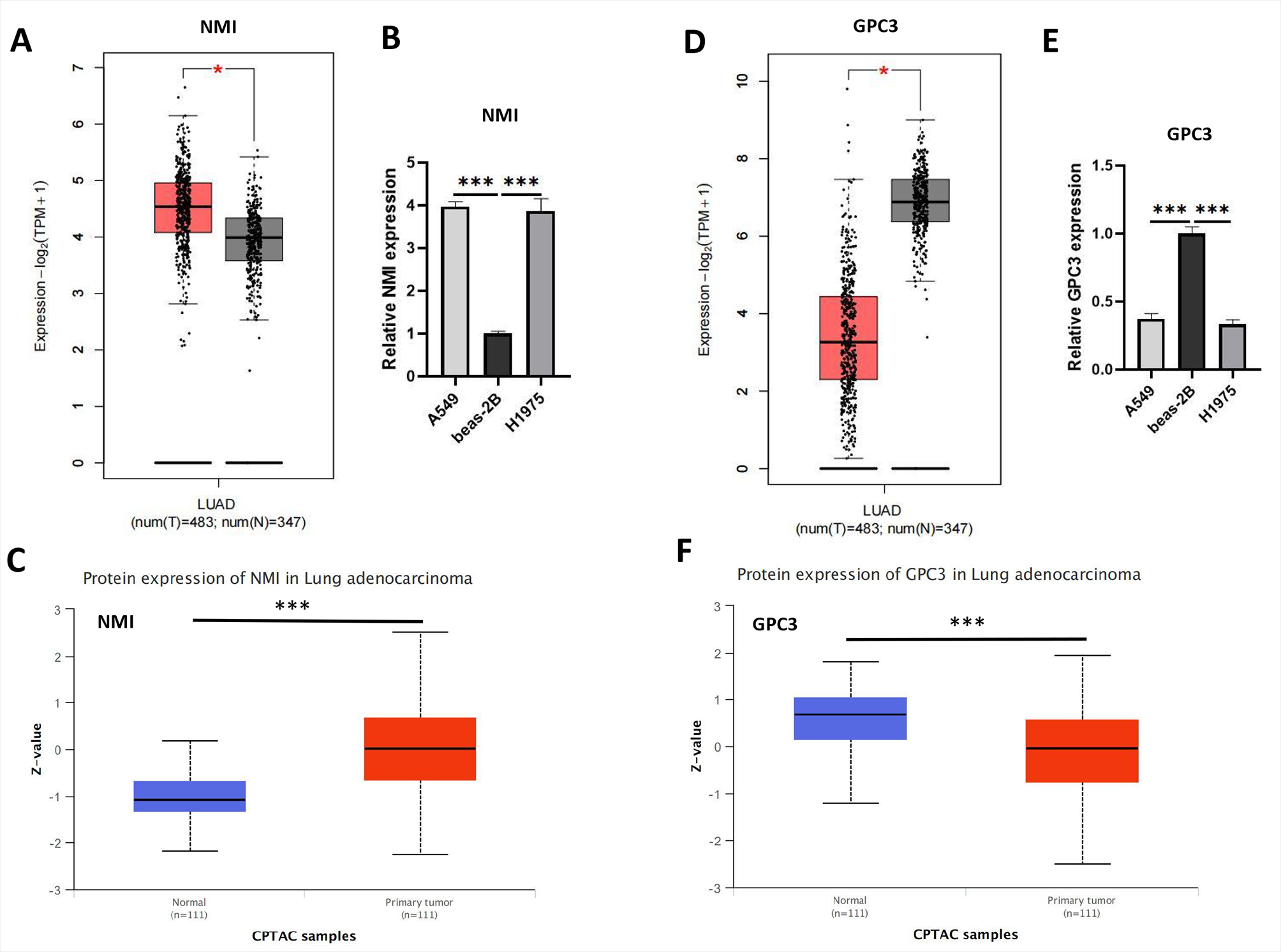


**Figure S5. Expression level of NMI and GPC3 in LUAD. (A)** Differential expression of NMI mRNA between LUAD tumor and normal tissues in GEPIA2 web (match TCGA normal and GTEx data) (http://gepia2.cancer-pku.cn/#analysis). **(B)** Differential expression of NMI mRNA between normal epithelial cells of the human lung (beas-2B) and lung cancer cells (A549 and H1975). **(C)** Differential expression of NMI protein between LUAD tumor and normal tissues in UALCAN portal (http://ualcan.path.uab.edu/analysis-prot.html). **(D)** Differential expression of GPC3 mRNA between LUAD tumor and normal tissues in GEPIA2 web (match TCGA normal and GTEx data) (http://gepia2.cancer-pku.cn/#analysis). **(E)** Differential expression of GPC3 mRNA between normal epithelial cells of the human lung (beas-2B) and lung cancer cells (A549 and H1975). **(F)** Differential expression of GPC3 protein between LUAD tumor and normal tissues in UALCAN portal (http://ualcan.path.uab.edu/analysis-prot.html).

**
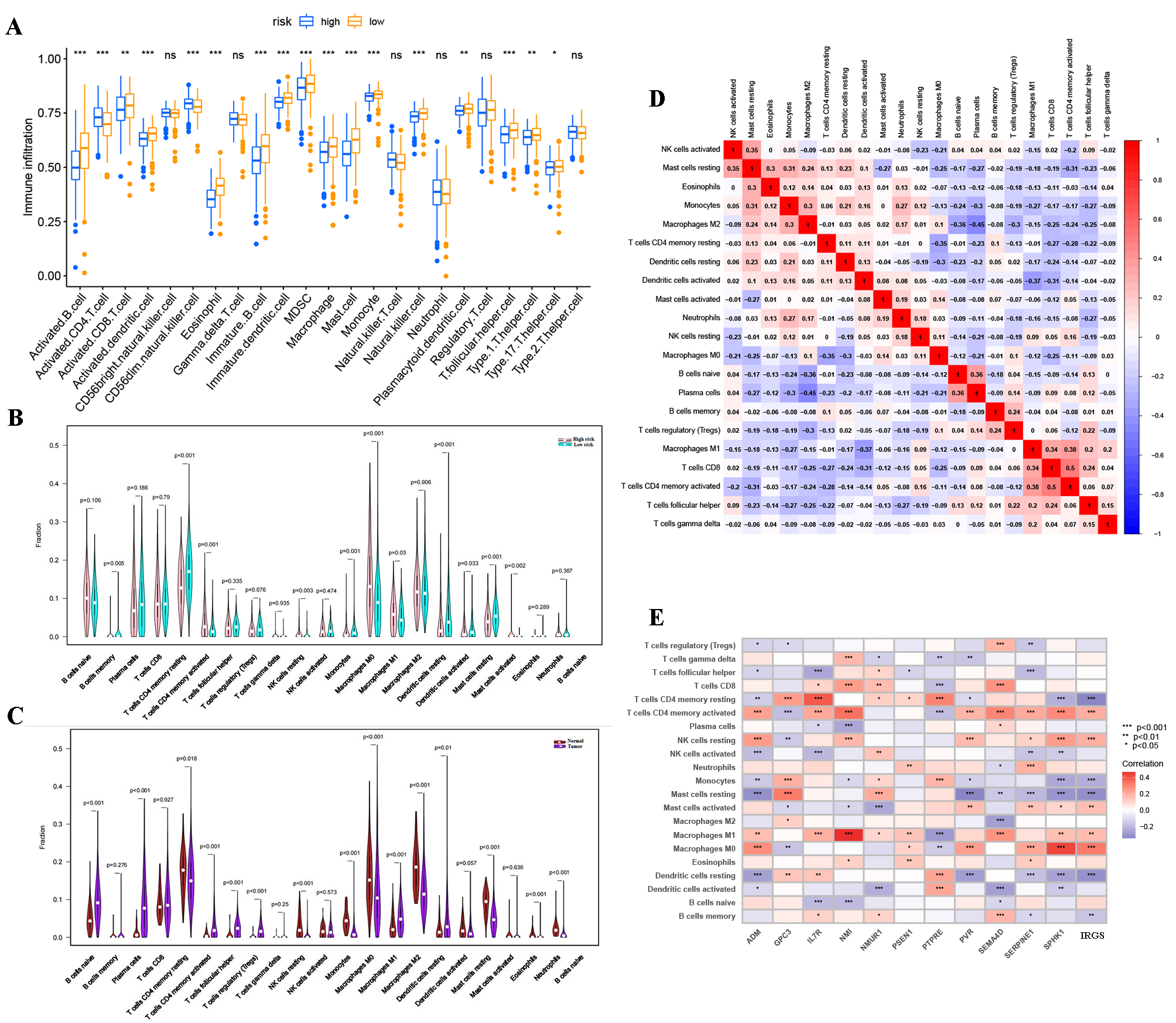
**

**Figure S6. Enriched biological pathways and TME characteristics in different subgroups.** (**A**) Comparison of tumor immune infiltrating cells based on ssGSEA algorithm in the high- and low-IRGS groups. The asterisks represented the statistical P-value (*P < 0.05; **P < 0.01; ***P < 0.001). (**B**) Tumor immune infiltrating cells based on CIBERSORT algorithm between different IRGS subgroups were analyzed and compared. (**C**) Comparison of tumor immune infiltrating cells based on CIBERSORT algorithm in the LUAD tumor and normal tissues. (**D**) Correlation between the infiltrating abundance of different immune cells in LUAD tumor tissues. (**E**) Correlation between the infiltrating abundance of immune cells using CIBERSORT algorithm and IRG signature gene expression as well as IRGS. (* represented p < 0.05; ** represented p< 0.01; *** represented p < 0.001).


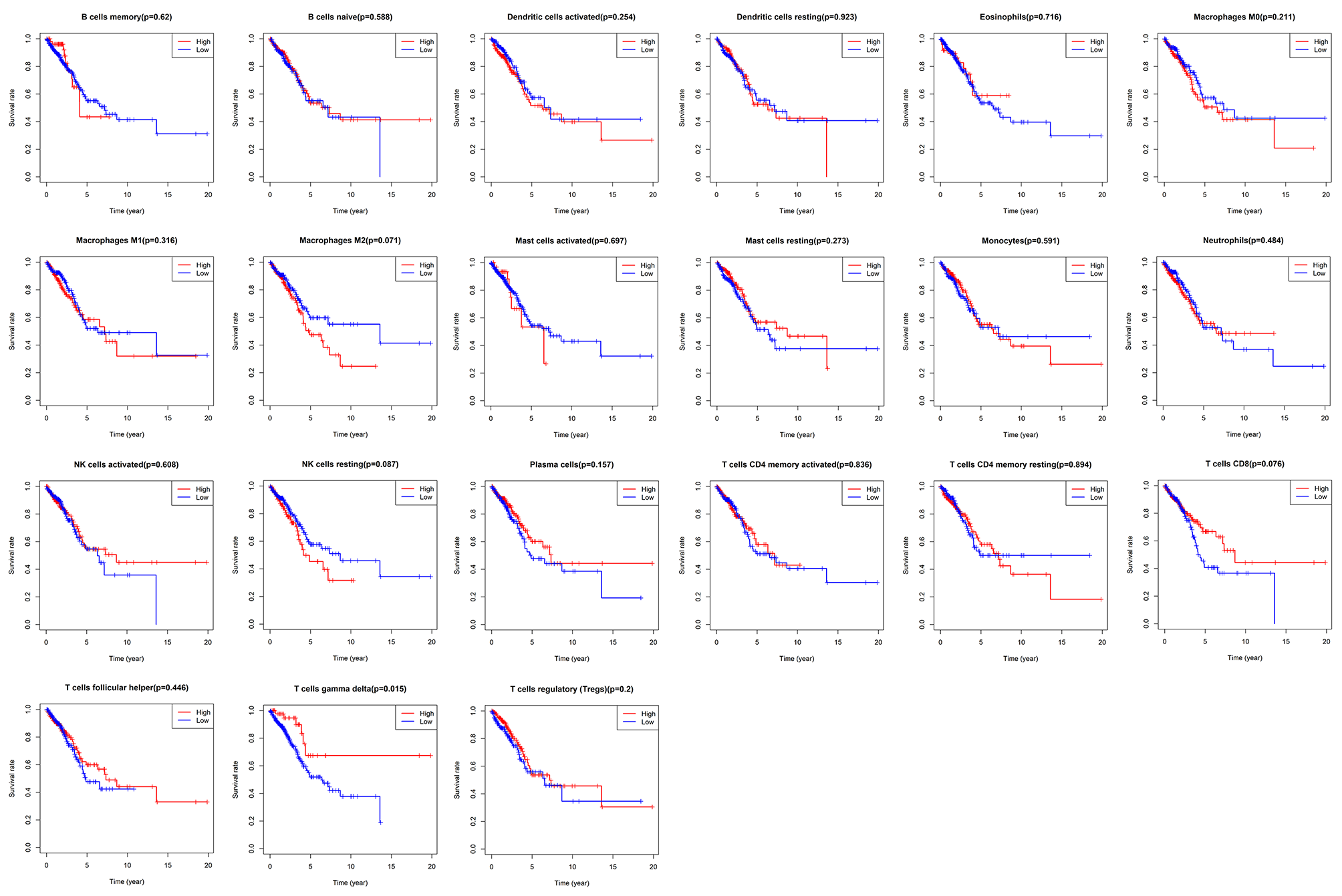


**Figure S7.** Kaplan-Meier survival analysis revealed the relationship between immune-infiltrating cells and patient survival.

**
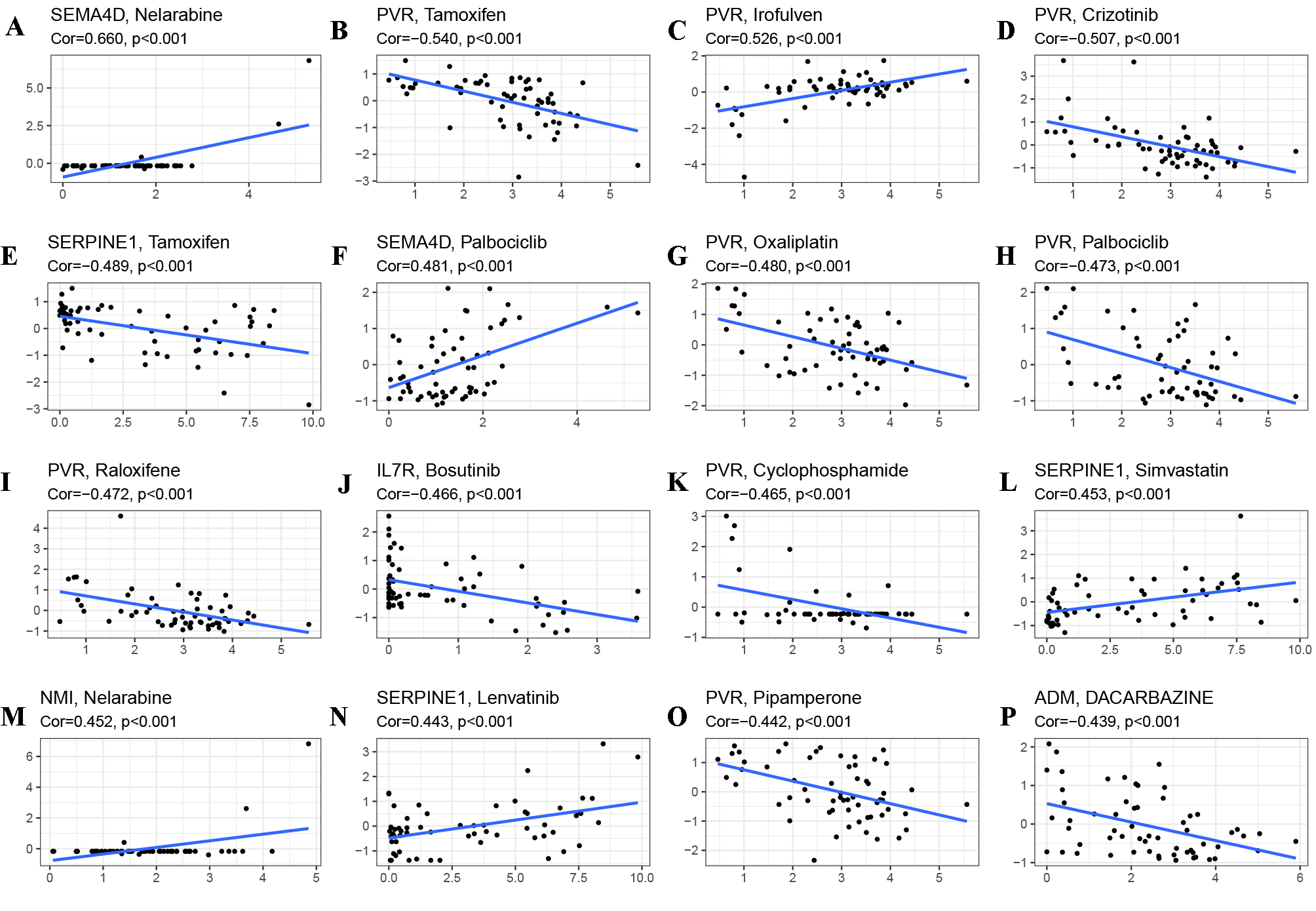
**

**Figure S8. Drug sensitivity analysis.** Correlation between the first 16 drugs (p values small to large) and the IRG signature genes.


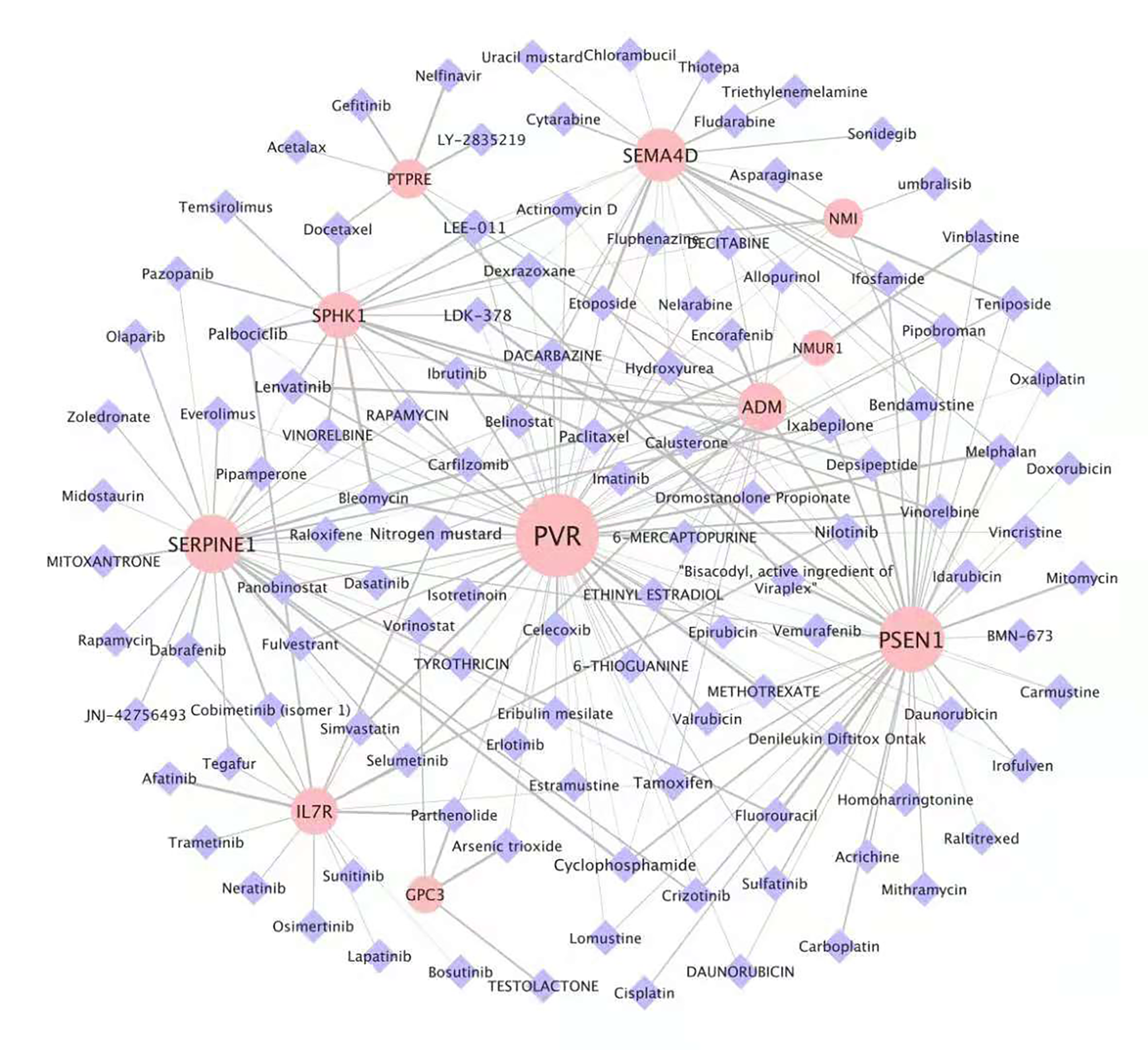


**Figure S9.** Network plots visualized the correlation between the 11 IRG signature genes and FDA approved drugs.
